# Artificial Intelligence and Complex Network Approaches Reveal Potential Gene Biomarkers for Hepatocellular Carcinoma

**DOI:** 10.3390/ijms242015286

**Published:** 2023-10-18

**Authors:** Antonio Lacalamita, Grazia Serino, Ester Pantaleo, Alfonso Monaco, Nicola Amoroso, Loredana Bellantuono, Emanuele Piccinno, Viviana Scalavino, Francesco Dituri, Sabina Tangaro, Roberto Bellotti, Gianluigi Giannelli

**Affiliations:** 1 Dipartimento Interateneo di Fisica M. Merlin, Università degli Studi di Bari Aldo Moro, Via G. Amendola 173, 70125 Bari, Italy; antonio.lacalamita@uniba.it (A.L.); ester.pantaleo@uniba.it (E.P.); roberto.bellotti@ba.infn.it (R.B.); 2 Sezione di Bari, Istituto Nazionale di Fisica Nucleare (INFN), Via A. Orabona 4, 70125 Bari, Italy; nicola.amoroso@uniba.it (N.A.); loredana.bellantuono@ba.infn.it (L.B.); sonia.tangaro@ba.infn.it (S.T.); 3 National Institute of Gastroenterology S. De Bellis, IRCCS Research Hospital, Via Turi 27, 70013 Castellana Grotte, BA, Italy; grazia.serino@irccsdebellis.it (G.S.); emanuele.piccinno@irccsdebellis.it (E.P.); viviana.scalavino@irccsdebellis.it (V.S.); francesco.dituri@irccsdebellis.it (F.D.); gianluigi.giannelli@irccsdebellis.it (G.G.); 4 Dipartimento di Farmacia-Scienze del Farmaco, Università degli Studi di Bari Aldo Moro, Via A. Orabona 4, 70125 Bari, Italy; 5 Dipartimento di Biomedicina Traslazionale e Neuroscienze (DiBraiN), Università degli Studi di Bari Aldo Moro, Piazza G. Cesare 11, 70124 Bari, Italy; 6 Dipartimento di Scienze del Suolo, della Pianta e degli Alimenti, Università degli Studi di Bari Aldo Moro, Via G. Amendola 165/a, 70126 Bari, Italy

**Keywords:** HCC diagnosis, complex networks, machine learning, Leiden algorithm, XAI methods

## Abstract

Hepatocellular carcinoma (HCC) is one of the most common cancers worldwide, and the number of cases is constantly increasing. Early and accurate HCC diagnosis is crucial to improving the effectiveness of treatment. The aim of the study is to develop a supervised learning framework based on hierarchical community detection and artificial intelligence in order to classify patients and controls using publicly available microarray data. With our methodology, we identified 20 gene communities that discriminated between healthy and cancerous samples, with an accuracy exceeding 90%. We validated the performance of these communities on an independent dataset, and with two of them, we reached an accuracy exceeding 80%. Then, we focused on two communities, selected because they were enriched with relevant biological functions, and on these we applied an explainable artificial intelligence (XAI) approach to analyze the contribution of each gene to the classification task. In conclusion, the proposed framework provides an effective methodological and quantitative tool helping to find gene communities, which may uncover pivotal mechanisms responsible for HCC and thus discover new biomarkers.

## 1. Introduction

Hepatocellular carcinoma (HCC) is one of the leading causes of cancer-related deaths worldwide, and the number of cases is constantly increasing [1]. Although there have been significant improvements in diagnosis and in therapeutic interventions, HCC remains associated with poor prognosis in patients with advanced disease [2,3]. An early and accurate diagnosis is crucial to improving the therapeutic effectiveness of HCC. Serum α-fetoprotein (AFP) is currently widely used as a diagnostic and prognostic biomarker for HCC; however, when used alone, the results are unsatisfactory [4,5]. The sensitivity of AFP in detecting HCC at an early stage is limited [6,7,8]. Several studies have demonstrated poor AFP specificity in diagnosing HCC, since AFP levels increase also in other disorders or benign liver conditions [9]. The suggested guidelines for HCC surveillance reported that serum AFP could be used as a marker along with abdominal ultrasonography only in high-risk populations to detect HCC at an early stage [10,11]. The multifactorial nature of HCC [12] makes it difficult to predict the diagnosis using a single biomarker. Therefore, the combination of multiple biomarkers is key to increasing the diagnostic accuracy of HCC. Other tumor markers, such as the des-gamma-carboxyprothrombin (DCP) and the Lens culinaris agglutinin-reactive fraction of AFP (AFP-L3), have been used to increase the diagnostic accuracy of AFP [13,14]. In addition to tumor markers, biomarkers of liver inflammation (aspartate amino transferase and alanine amino transferase), fibrosis (platelet count), liver function (total bilirubin and albumin), and the hepatitis virus status are widely used in clinical practice [15,16]. However, due to the aforementioned limitations of these biomarkers for early diagnosis of HCC, there is an urgent need for the development of more sensitive diagnostic methods, the use of novel biomarkers, and the construction of prognostic models in order to increase the survival time of patients.

Microarray technology has enabled simultaneous measurement of the expression level of thousands of genes in a sample in a single experiment [17]. These gene expression profiles can be used to identify biomarkers or networks of genes that are dysregulated in cancerous versus normal samples [18]. However, microarray datasets typically have a small sample size (number of individuals or samples) but high dimensions (number of genes). If microarray data are employed to train machine learning classification algorithms to discriminate patients and controls, this imbalance typically causes overfitting issues. Designing efficient algorithms that can reduce the initial gene pool, consisting of thousands of genes, to a smaller set containing hundreds or tens of them; it is therefore critical to improve the prediction accuracy. 

The aim of our study is to develop a supervised learning framework to classify HCC patients vs. controls using gene expression data from microarray samples. More specifically, we used two microarray experiments comparing tumor tissues of HCC patients with adjacent normal tissues. We constructed a complex network in which genes correspond to nodes and their connections are weighted according to the strength of their co-expression relations [19,20]. Then, we performed a feature selection step, which is essential to developing a robust classification algorithm, using artificial intelligence methods coupled with complex network algorithms. In short, we applied hierarchical community detection using the Leiden algorithm [21], a community detection method that is especially useful when a network has both positive and negative links, as in our case. We identified 46 communities of genes, which we further filtered using the Boruta feature selection method [22]. Twenty of these communities could discriminate between healthy and cancerous samples with an accuracy exceeding 90%. We validated these communities on an independent dataset; eight of them still provided satisfactory classification performances, and two of them reached an accuracy of more than 80%. Finally, we focused on two communities, selected because they were enriched with relevant biological functions, and on these we applied an explainable artificial intelligence approach [23,24] to analyze the contribution of each of their genes to the classification task. 

The paper is organized as follows. In the Section 2, we present an overview of the methodology adopted and report the outcome with associated figures and tables. In the Section 3, we comment on our results and underline some limitations of our work. In the Section 4, we provide details about the input data and the normalization procedure (Section 4.1 “Data Sources”), and then we describe the methods used (subsections include Section 4.2 “Hierarchical community detection”, Section 4.3 “Feature selection”, Section 4.4 “Machine learning scheme”, Section 4.5 “XAI analysis”, and Section 4.6 “Gene Set Enrichment Analysis”). 

## 2. Results

We downloaded gene expression data on liver tissue from 152 patients who underwent hepatic resection. The data consisted of 152 tumor samples and 91 adjacent liver tissues, as well as 14 adjacent liver tissues obtained from patients with colorectal cancer metastasis who had not received chemotherapy (see Section 4 “Material and methods” for more details). 

**Figure 1:**
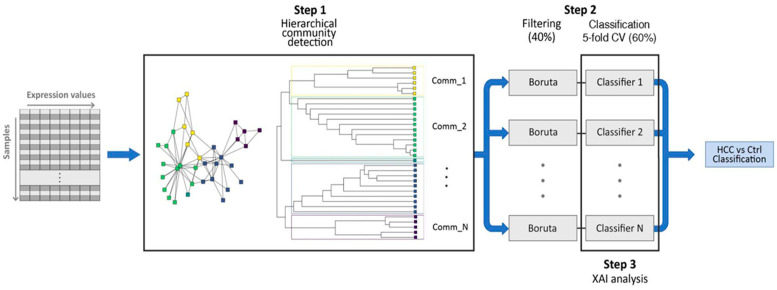
Schematic representation of the workflow in our study. After detecting the communities with highly correlated gene expression profiles through a procedure based on the Leiden algorithm, we estimated the most informative genes with the Boruta algorithm and used them to feed a random forest model inside a 5-fold cross-validation scheme.

Our computational pipeline consisted of three main steps: (i) a hierarchical community detection phase, in which we applied the Leiden algorithm to find stable gene communities within the gene co-expression network; (ii) an additional learning phase, in which we focused on each community, selected a subset of genes with the Boruta method, and fed this subset to a random forest (RF) algorithm in a 5-fold cross validation framework to obtain the classification of HCC vs. normal samples; and (iii) an explainable artificial intelligence (XAI) phase, in which we quantified the impact of each gene at the community scale on the classifier’s predictions by applying Shapley (SHAP) values. Finally, we validated the model using an independent gene expression dataset. A schematic overview of our workflow is displayed in Figure 1. 

The original dataset is composed of samples belonging to three different classes: HCC, Normal and Peritumoral samples. We implemented an unsupervised analysis to see if there was a statistically significant difference between Normal and Peritumoral samples.

Firstly, we determined the optimal number of clusters using two quantities, the Within Sum of Square (WSS) and the Silhouette coefficient (SC). From Figure S1 we can see that the optimal number of clusters is 2 with both WSS (left) and SC (right) (using the elbow method and the maximum value, respectively). Once we set the number of clusters, we applied a k-means clustering algorithm with k = 2, displayed in Figure S2. In Table S1, we presented cluster cardinalities and compared original labels vs k-means labels in a contingency matrix. These results confirm that 95.24% of Normal + Peritumoral samples belong to the same cluster, therefore there is no statistically significant difference between Normal and Peritumoral samples.

**Figure 2:**
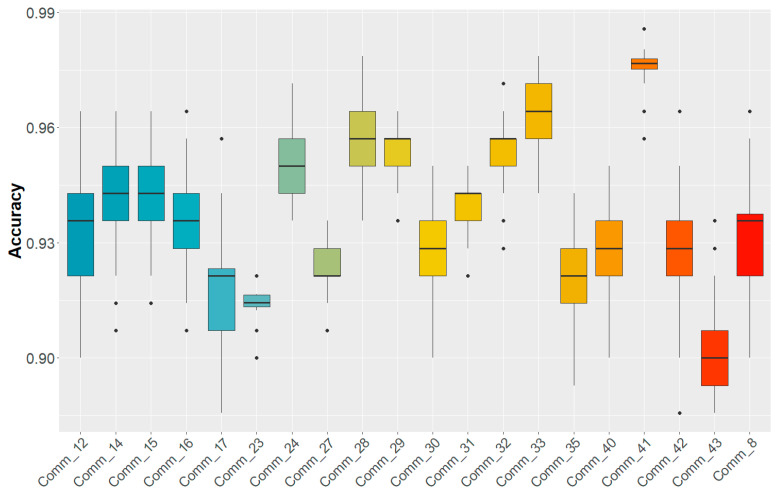
Boxplot of the accuracy values of the 20 best communities averaged over 100 5-fold cross-validation rounds.

From the hierarchical community detection procedure, we derived 46 stable communities. As shown in Table 1, 20 of these gene communities gave a robust prediction of the disease (accuracy exceeding 90%). Our choice of the random forest algorithm was suggested by a previous study on microarrays of cancerous tissues [25], where we found that RF was one of the best performing methods, as well as being the easiest to tune and the lightest in terms of computational burden. Table 1 also reports area under the curve (AUC), F1 Score, and Log Loss. In Figure 2 we display the boxplot of the accuracy values of the 20 top communities. The distributions were computed through a 5-fold cross validation procedure repeated 100 times. In the Supplementary Materials, we list the complete set of genes in each of these communities. 

In order to validate our results, we tested the performance of the 20 identified communities on an independent dataset. We report our results in Table 2, showing that eight of these communities still had satisfactory accuracy values, especially for an independent test; in particular, Comm_29 and Comm_32 reached an accuracy, AUC, and F1 Score of more than 80%.

After close biological inspection of the set of 20 communities, we identified two communities, namely, Comm_29 and Comm_41, which were particularly interesting as they were enriched with relevant biological functions. It is worth noting that the first of these two communities (Comm_29) is the second best performing on the test set (as already mentioned),while Comm_41 was one of the top performing communities on the test set (with 74.5% accuracy).

### 2.1. XAI Analysis

**Figure 3:**
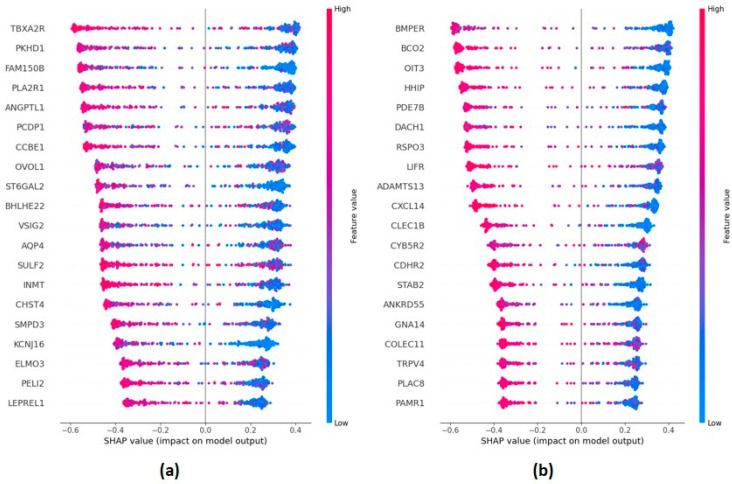
SHAP values of the 20 most important features in the classification of healthy vs. HCC samples for communities Comm_29 (panel (**a**)) and Comm_41 (panel (**b**)). Each point in the same row corresponds to a different patient.

We conducted a study of explainability by applying the Shapley (SHAP) algorithm [22,23] on a subset of the 20 communities, namely, Comm_29 and Comm_41, because they were enriched with relevant biological functions. In Figure 3, we display the resulting SHAP plots, which show the direction of the relationship between the expression of individual genes and the classification outcome. Each row corresponds to a gene within the considered community and includes a distribution of points, each representing a prediction on a subject. The horizontal axis reports the SHAP-values, quantifying the impact of features, i.e., gene expression values, on the different predictions provided by the machine learning classifier. Positive SHAP-values are indicative of the contribution of the specific gene expression value to a positive diagnosis (HCC), while negative SHAP-values are indicative of a contribution to a negative diagnosis (no HCC). In each row, instances with higher and lower values of the gene expression feature are shown in red and blue, respectively. This reveals that a higher expression of gene TBXA2R, for example, is associated with HCC samples, while a lower expression is associated with healthy samples.

### 2.2. Pathways Analysis

Functional enrichment analysis with GSEA [26] of genes in Comm_29 and Comm_41 revealed that they are involved in hallmarks of the matrisome and immune microenvironment, which are two important features of the HCC pathogenesis (FDR < 0.05; Tables S2 and S3 in the Supplementary Materials). 

## 3. Discussion

HCC management is seriously challenged by the lack of biomarkers that could enable a reliable diagnosis and prognosis of HCC [27]. The use of serum enzymes, such as AFP, DCP, and AFP-L3, has been demonstrated to be more effective than other methods in monitoring tumor progression [28]. However, the use of serum enzymes lacks the sensitivity and specificity required for a reliable diagnosis and prognosis of HCC. 

Recent advances in multi-omics technologies in HCC studies have led to the discovery of new candidate biomarkers for diagnosis and prognosis [29]. Technologies such as microarrays or next-generation sequencing can provide useful tools with which to identify new genes or mechanisms aiding to making an early discrimination of a pathological state. In this paper, using microarray gene expression data from GEO, we developed a supervised learning framework in order to classify HCC patients and controls. 

More specifically, we analyzed microarray gene expression data of HCC tumor and adjacent liver tissues from the dataset GSE20295. Through a hierarchical community detection phase based on the Leiden algorithm, we found 46 stable communities. With a machine learning procedure combining RF, Boruta, and 5-fold cross-validation, among these 46 communities, we were able to identify 20 gene communities that could discriminate between healthy and HCC patients, with a mean accuracy exceeding 90%, as shown in Figure 2.

We validated our findings on an independent liver microarray dataset, GSE54236, and confirmed that two communities, namely, Comm_29 and Comm_32 (see Supplementary Materials), could distinguish between healthy subjects and HCC patients with an accuracy of more than 80%.

Functional enrichment analysis of genes revealed that two communities, namely, Comm_29 and Comm_41, are involved in two main hallmarks of HCC, i.e., the matrisome and the immune microenvironment, respectively. The matrisome includes extracellular matrix (ECM) molecules (collagens, glycoproteins, and proteoglycans) and ECM-associated members (ECM regulators, ECM-affiliated proteins, and secreted factors) [30]. Matrisome remodeling is one of the main features of liver fibrosis and occurs in carcinogenesis during cell proliferation, migration, or invasion [31]. Several studies have reported that the tumor microenvironment (TME) plays a critical role in HCC onset, progression, and outcome [32,33,34]. 

The results obtained in our study can help identify potential new markers for the diagnosis of HCC. However, our study has several limitations. The sample size of the experimental data we used is relatively small, so other studies are necessary in order to validate and improve the identification of genes in a larger multicenter prospective cohort of patients and controls. Furthermore, we are aware that machine learning algorithms are less effective when the training datasets contain a small number of observations. An improvement to our work would be the application of the same models to a larger training set. Moreover, the results of our study were obtained from tissue samples, which is still an invasive procedure. Further research is needed in order to determine whether the identified genes can be detected in blood so as to promote a non-invasive diagnosis and prognosis.

In conclusion, in our study, we identified two sets of genes expressed in liver samples that can distinguish HCC patients from healthy subjects with high accuracy. The robustness of our results, which were obtained from a cross-validation procedure and validated on an independent dataset, suggests they may have a potential clinical significance as a means of identifying biomarkers and new therapeutic targets.

## 4. Materials and Methods

### 4.1. Data Sources 

Two microarray datasets, GSE102079 and GSE54236, were downloaded from the GEO database (http://www.ncbi.nlm.nih.gov/geo/; accessed on 9 January 2023). 

Dataset GSE102079 contains gene expression data of the liver tissue of 152 patients who underwent hepatic resection from the GPL570 Affymetrix Human Genome U133 Plus 2.0 Array. Specifically, this set contains 152 tumor and 91 adjacent liver tissues from HCC patients and 14 adjacent liver tissues obtained from patients with metastasis of colorectal cancer who had not received chemotherapy. 

Dataset GSE54236 contains gene expression data of 156 samples of 78 HCC tumor tissues and 78 adjacent non-tumor tissues from the GPL6480 Agilent-014850 Whole Human Genome Microarray. This dataset was used for independent testing.

Raw data were normalized using robust multiarray analysis (RMA) [35]; this method implements a background correction of the original data; then, a log2 transformation and finally a quantile normalization.

For our analysis, we used the R framework version 4.2.2 [36] with packages oligo version 1.62.2 [37] to read CEL files; affy version 1.76.0 [38] to perform RMA; factoextra version 1.0.7 [39] to evaluate silhouette; amap version 0.8-19 [40] to perform K-means clustering; Boruta version 8.0.0 [22]; random forest version 4.7-1.1 [41]; treeshap version 0.1.1 [42]; and igraph version 1.4.1 [43]. For the Leiden algorithm, we used igraph function *cluster_leiden* with parameters resolution_parameter = 1, objective_function = “modularity”, n_iterations = 200, and beta = 0.05; and for evaluating the mutual information, we used function *compare* with method = “nmi”. 

### 4.2. Hierarchical Community Detection

We implemented a hierarchical community detection workflow based on iterative applications of the Leiden algorithm [21] to find groups (or communities) of genes with highly-correlated gene expression profiles. The Leiden algorithm identifies an optimal partition of the network, which maximizes positive-weight connections within communities and negative-weight connections across communities, starting from a random configuration consisting of an arbitrary number of communities to which nodes are arbitrarily assigned. Due to the inherent randomness of the Leiden community detection algorithm, it is necessary to check that the partition it provides is stable compared with the initial conditions. The method we employed to quantify the stability of the community detection outcome is discussed below. Communities of the optimal partition were independently used to implement machine learning algorithms for the classification of HCC vs. healthy samples.

Because discovering meaningful biological knowledge from communities with more than 100 genes is challenging [44,45], we iteratively applied the Leiden algorithm on the whole co-expression network until we obtained communities with less than 100 genes. We also ignored communities with less than 4 elements. Specifically, the communities found in the first step were considered to be second-level co-expression subnetworks, to which the Leiden algorithm was independently applied; this hierarchical process was iterated at the next levels for each community in a given partition until the size of its further subdivisions became smaller than 100. 

The Leiden algorithm, when applied iteratively, converges on a partition in which all subsets of the obtained communities are locally optimally assigned. At each step of the hierarchical community detection process, the most stable partition was found by tuning two internal parameters of the Leiden algorithm, namely, the resolution γ and the level of randomness β, while keeping the other parameters fixed to their default values. For each configuration (γ,β) of the considered internal parameters, the stability of the community detection outcome was evaluated by performing *L* = 100 runs of the Leiden algorithm, each corresponding to a different seed of the pseudorandom number generator and to a different initial arbitrary assignment of nodes to groups. For the *j*-th run (j=1,…,L), the algorithm returned a partition $p_{j}$, and the most recurring (majority) partition over all *L* runs was determined via majority voting. This partition was considered acceptable provided that it was simultaneously, stable, nontrivial, and not fragmented. Below we describe each of these conditions in detail.

The stability criterion was based on the similarity between partitions ${\left\{p_{j}\right\}}_{j=1,\ldots ,L}$ obtained in the *L* runs and corresponding to the different random initial conditions. Specifically, this similarity was evaluated through the average normalized mutual information:

(1) $$\langle NMI\rangle =\frac{2}{L\left(L-1\right)}\sum_{a=1}^{L-1}\sum_{b=a+1}^{L}NMI\left(p_{a},p_{b}\right),$$

where $NMI\left(p_{a},p_{b}\right)$ is the normalized mutual information between a given pair $\left(p_{a},p_{b}\right)$ of partitions, and L(L−1)/2 is the number of distinct pairs. The majority partition was considered stable only if 〈NMI〉 ≥0.80, a condition related to the general uniformity of the partitions returned by the different runs. 

Moreover, the most recurring partition returned by the Leiden algorithm must be nontrivial, i.e., not consist of a single community, coinciding with the whole network or subnetwork on which the Leiden algorithm was applied.

Finally, to avoid excessive fragmentation, the most recurrent partition over *L* runs must not include communities whose number of nodes is less than 5% of the whole network cardinality. 

In cases where multiple configurations (γ,β) of the internal parameters of the Leiden algorithm provided a majority partition that satisfied the aforementioned requirements, the one with the highest 〈NMI〉 was chosen and identified as the outcome of the hierarchical community detection at the considered level.

### 4.3. Feature Selection

Following the gene community detection procedure explained in the previous section, as the number of input features (genes) still exceeded the number of samples, we filtered the genes in each community further using a wrapper method known as Boruta [22]. This method aims to eliminate noise and redundant data by selecting only those features that enhance the performance of the learning model. 

Boruta, a robust and efficient supervised feature selection algorithm, is based on the random forest method. It leverages the principles of random forest (RF) (see the following section), where random perturbations in the system and the randomization of the training samples mitigate the negative effects caused by the random fluctuations and correlations inherent to the learning model. Essentially, Boruta improves independent classification and regression trees (CART) by generating surrogate features, referred to as shadow features, through shuffling of the original feature values. It then compares the importance of these shadow features with that of the original features within the model. In simple terms, Boruta employs these shadow attributes as reference values to quantify the importance of an attribute. 

We applied the Boruta algorithm to each community selected by the gene community procedure. To avoid data leakage, we used 40% of the dataset for feature selection and the remaining 60% for classification.

### 4.4. Machine Learning Scheme

Gene community detection and feature selection allowed us to achieve a significant reduction in the number of features. The remaining genes were used as input to the random forest (RF) algorithm. We developed an RF model for each community. 

RF [46] is a highly popular machine learning algorithm because its parameters are easy to tune, particularly the two main parameters: (i) the number of trees, denoted as M; and (ii) the number of randomly selected features at each split, denoted as s. The RF structure consists of an ensemble of CART classification trees, which are grown using a bootstrap procedure. The randomization process during training makes RF robust to overfitting and generates trees that exhibit low mutual correlation. In this study, we implemented a standard configuration with M = 300 trees and s = |S|, where S represents the set input features and |.| represents the cardinality. 

To enhance the robustness of our approach, we applied a 5-fold cross-validation scheme that we repeated 100 times. We carried out this procedure on the subjects. 

### 4.5. XAI Analysis

Explainable artificial intelligence (XAI) procedures aim at improving machine learning transparency and interpretability, especially when applied to real-world scenarios [47,48,49,50,51,52]. As opposed to traditional approaches that focus on informativeness, quantified by performance metrics and uncertainties [53,54,55], a XAI framework attempts to combine informativeness with generalization, namely, the reliability of predictions on previously unseen data and transparency, aimed at making model decisions as easy to understand as possible [56,57].

In this study, we used a SHAP local explanation algorithm to determine the importance of features for the classification of HCC vs. normal samples. This algorithm is a local, model-agnostic post hoc explainer, based on the Shapley values concept borrowed from cooperative game theory [23,24]. For each sample, the SHAP algorithm learns local interpretable linear models and quantifies the contribution of each feature to the prediction given by the model for that sample. The computation of a SHAP value for a given feature is based on the difference between the model prediction when that feature is considered or not considered, averaging over all possible subsets of features. The model is thus retrained on all the possible feature subsets *F* of the complete feature set *S* (F⊆S). Calling $f_{x}\left(F\right)$ the model prediction for instance *x* when the subset *F* does not include the *j*-th feature, and $f_{x}\left(F\cup j\right)$ the prediction when the *j*-th feature is added, the difference $f_{x}\left(F\cup j\right)-f_{x}\left(F\right)$ quantifies the marginal contribution of the *j*-th feature. The SHAP value of the j-th feature for the instance x is then evaluated as

(2) $$SHAP_{j}\left(x\right)=\sum_{F\subseteq S-\left\{j\right\}}\frac{\left|F\right|!\left(\left|S\right|-\left|F\right|-1\right)!}{\left|S\right|!}[f_{x}\left(F\cup j\right)-f_{x}\left(F\right)],$$

with |F|! is the number of permutations of features in the subset *F*; (|S|−|F|−1)! is the number of permutations of features in the subset S−(F∪{j}); and |S|! is the total number of feature permutations [23].

### 4.6. Gene Set Enrichment Analysis

Gene set enrichment analysis (GESA) was performed with GSEA software version MSigDB 2023.1 [26] using the hallmark gene sets of the Molecular Signature Database gene set, canonical pathways, and gene ontology. An FDR value threshold of 0.05 was used to select significant hallmarks.

## Figures and Tables

**Table 1:** Number of genes, accuracy, AUC, F1 Score, and Log Loss of the classifiers obtained using the 46 stable communities. Results for each classifier were obtained after 100 5-fold cross-validation rounds.

Community	Cardinality	Accuracy (%)	AUC (%)	F1 Score (%)	Log Loss
Comm_1	3	59.61 ± 3.06	57.64 ± 3.15	48.61 ± 4.12	5.64 ± 2.31
Comm_2	16	87.28 ± 1.29	81.47 ± 2.10	77.77 ± 2.70	−4.11 ± 1.26
Comm_3	21	77.89 ± 2.05	78.55 ± 2.01	74.16 ± 2.54	−2.67 ± 1.58
Comm_4	7	73.28 ± 2.45	92.37 ± 1.41	91.32 ± 1.62	−6.31 ± 0.56
Comm_5	24	87.28 ± 1.57	85.22 ± 1.85	82.46 ± 2.20	−1.34 ± 1.06
Comm_6	20	83.37 ± 1.82	93.58 ± 1.38	92.66 ± 1.65	−5.76 ± 0.95
Comm_7	15	86.66 ± 2.03	93.59 ± 1.18	92.63 ± 1.37	−5.58 ± 0.72
Comm_8	33	93.22 ± 1.30	92.79 ± 1.29	91.63 ± 1.46	−5.16 ± 0.52
Comm_9	21	70.59 ± 2.06	90.79 ± 1.33	89.41 ± 1.62	−5.94 ± 0.80
Comm_10	23	83.06 ± 1.92	82.66 ± 1.87	79.35 ± 2.32	−3.14 ± 1.14
Comm_11	20	80.13 ± 1.96	88.85 ± 1.98	87.01 ± 2.42	−5.50 ± 1.13
Comm_12	49	93.23 ± 1.19	86.64 ± 1.29	84.20 ± 1.55	−2.67 ± 1.13
Comm_13	23	85.69 ± 1.71	70.49 ± 2.26	63.97 ± 2.93	−0.18 ± 1.63
Comm_14	34	94.17 ± 1.30	87.12 ± 1.50	84.80 ± 1.80	−3.53 ± 1.06
Comm_15	27	94.13 ± 1.07	87.33 ± 1.64	84.86 ± 1.88	−0.24 ± 1.07
Comm_16	34	93.32 ± 1.11	91.42 ± 0.46	89.69 ± 0.54	−2.55 ± 0.41
Comm_17	30	91.74 ± 1.25	95.54 ± 0.81	94.29 ± 0.97	−2.87 ± 0.74
Comm_18	18	83.88 ± 1.75	84.81 ± 1.28	81.97 ± 1.50	−0.29 ± 1.10
Comm_19	32	89.92 ± 1.79	88.47 ± 0.89	86.29 ± 1.04	−1.96 ± 0.74
Comm_20	8	72.39 ± 2.18	91.96 ± 0.63	90.53 ± 0.78	−4.14 ± 0.59
Comm_21	31	87.91 ± 1.39	95.44 ± 0.88	94.64 ± 1.08	−5.07 ± 0.78
Comm_22	44	87.29 ± 1.57	95.78 ± 0.59	94.57 ± 0.64	−3.03 ± 0.28
Comm_23	40	91.47 ± 0.45	76.07 ± 2.14	70.90 ± 2.83	−2.47 ± 2.14
Comm_24	29	95.19 ± 0.83	93.12 ± 1.12	91.41 ± 1.28	−1.41 ± 0.89
Comm_25	24	85.07 ± 1.19	94.36 ± 0.67	92.89 ± 0.75	−2.25 ± 0.50
Comm_26	36	88.72 ± 0.88	95.18 ± 0.87	94.31 ± 1.02	−4.90 ± 0.56
Comm_27	44	92.34 ± 0.66	96.01 ± 0.94	95.34 ± 1.09	−5.40 ± 0.64
Comm_28	40	95.64 ± 0.89	74.93 ± 1.69	69.67 ± 2.17	−1.18 ± 1.21
Comm_29	51	95.44 ± 0.52	92.00 ± 1.21	90.38 ± 1.34	−2.84 ± 0.47
Comm_30	37	92.69 ± 1.09	80.38 ± 1.63	76.84 ± 1.90	1.57 ± 1.08
Comm_31	31	93.99 ± 0.63	78.55 ± 1.51	74.33 ± 1.88	−1.26 ± 1.10
Comm_32	41	95.37 ± 0.82	82.98 ± 2.21	79.80 ± 2.66	−1.06 ± 1.34
Comm_33	41	96.23 ± 0.87	89.45 ± 0.91	87.49 ± 1.11	−2.81 ± 0.89
Comm_34	23	76.56 ± 1.61	72.10 ± 2.62	66.68 ± 3.31	2.02 ± 1.87
Comm_35	32	92.06 ± 1.05	92.62 ± 1.08	91.23 ± 1.30	−3.79 ± 0.85
Comm_36	26	80.70 ± 1.58	97.79 ± 0.40	97.22 ± 0.56	−5.04 ± 0.51
Comm_37	33	79.78 ± 1.47	92.30 ± 1.41	91.02 ± 1.65	−4.86 ± 0.90
Comm_38	4	83.65 ± 2.05	89.74 ± 1.01	87.91 ± 1.20	−3.71 ± 0.65
Comm_39	36	89.79 ± 0.96	80.23 ± 1.54	76.51 ± 1.83	−0.46 ± 1.24
Comm_40	30	92.84 ± 1.07	76.38 ± 1.66	71.53 ± 2.03	−1.43 ± 1.36
Comm_41	53	97.71 ± 0.48	67.03 ± 2.39	60.24 ± 3.02	2.72 ± 1.86
Comm_42	38	92.81 ± 1.29	86.12 ± 1.74	83.62 ± 2.15	−4.55 ± 0.90
Comm_43	35	90.27 ± 0.95	81.96 ± 1.94	78.44 ± 2.46	−3.61 ± 1.34
Comm_44	22	81.07 ± 1.54	85.34 ± 2.25	82.65 ± 2.80	−4.83 ± 1.00
Comm_45	20	77.91 ± 1.67	93.23 ± 1.29	91.81 ± 1.54	−3.17 ± 1.07
Comm_46	6	68.62 ± 2.33	68.84 ± 2.25	62.14 ± 3.06	1.21 ± 1.44

**Table 2:** Accuracy, AUC, F1 Score, and Log Loss of the 20 best classifiers tested on the independent dataset.

Community	Cardinality	Accuracy (%)	AUC (%)	F1 Score (%)	Log Loss
Comm_8	33	51.55	51.25	4.88	16.73
Comm_12	49	59.01	58.80	37.74	14.16
Comm_14	34	61.49	61.28	41.51	13.30
Comm_15	27	66.46	66.33	57.81	11.58
Comm_16	34	70.81	70.73	66.19	10.08
Comm_17	30	50.93	50.64	7.06	16.95
Comm_23	40	50.31	50.00	0.00	17.16
Comm_24	29	50.31	50.00	0.00	17.16
Comm_27	44	63.98	63.82	51.67	12.44
Comm_28	40	68.94	68.78	57.63	10.73
Comm_29	51	81.37	81.38	81.71	6.44
Comm_30	37	50.93	50.62	2.47	16.95
Comm_31	31	71.43	71.53	75.27	9.87
Comm_32	41	82.61	82.62	82.72	6.01
Comm_33	41	70.81	70.67	62.40	10.08
Comm_35	32	74.53	74.63	77.84	8.80
Comm_40	30	71.43	71.45	72.29	9.87
Comm_41	53	74.53	74.65	78.53	8.80
Comm_42	38	51.55	51.26	7.14	16.73
Comm_43	35	50.31	50.00	0.00	17.16

## Data Availability

Not applicable.

## Supplementary Materials

The supporting information can be downloaded at https://www.mdpi.com/article/10.3390/ijms242015286/s1.
