# Supplementary material for: Artificial Intelligence and Complex Network Approaches Reveal Potential Gene Biomarkers for Hepatocellular Carcinoma
Source: Int J Mol Sci. 2023 Oct 18;24(20):15286. doi: 10.3390/ijms242015286 (PMC10607580; doi:10.3390/ijms242015286)
Supplement: Supplementary file 1 [file ijms-24-15286-s001.zip › ijms-2632929-supplementary.pdf]

Supplementary Materials

Exploratory analysis - Unsupervised clustering analysis

The original dataset is composed of samples belonging to three different classes: HCC, Normal and Peritumoral samples. We implemented an unsupervised analysis in order to see if there was a statistically significant difference between Normal and Peritumoral samples.

Firstly, we determined the optimal number of clusters using two quantities, the Within Sum of Square (WSS) and the Silhouette coefficient (SC). From Figure S1 we can see that the optimal number of clusters is 2 with both WSS (left) and SC (right) (using the elbow method and the maximum value, respectively).

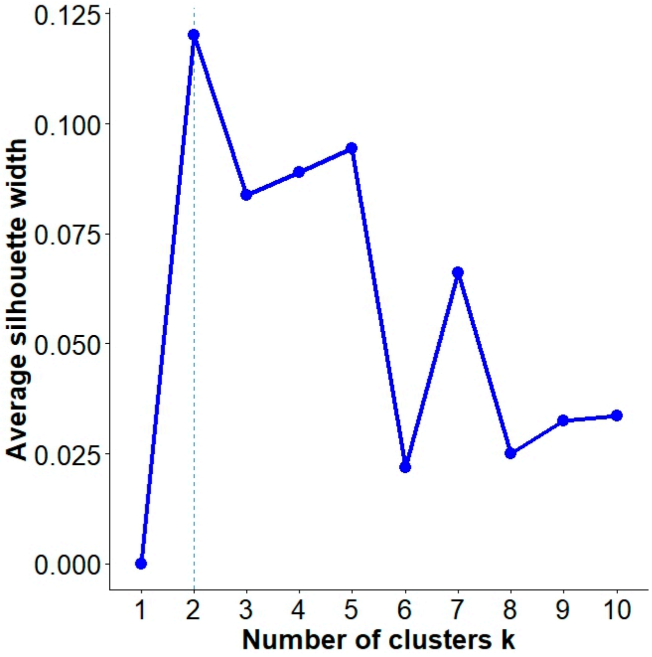

Figure S1: WSS (left) and SC (right) values for different numbers of clusters.

Once we set the number of clusters, we applied a k-means clustering algorithm with k=2, displayed in Figure S2. In Table S1, we presented cluster cardinalities and compared original labels vs k-means labels in a contingency matrix. These results confirm that 95.24% of Normal+Peritumoral samples belong to the same cluster, therefore there is no statistically significant difference between Normal and Peritumoral samples.

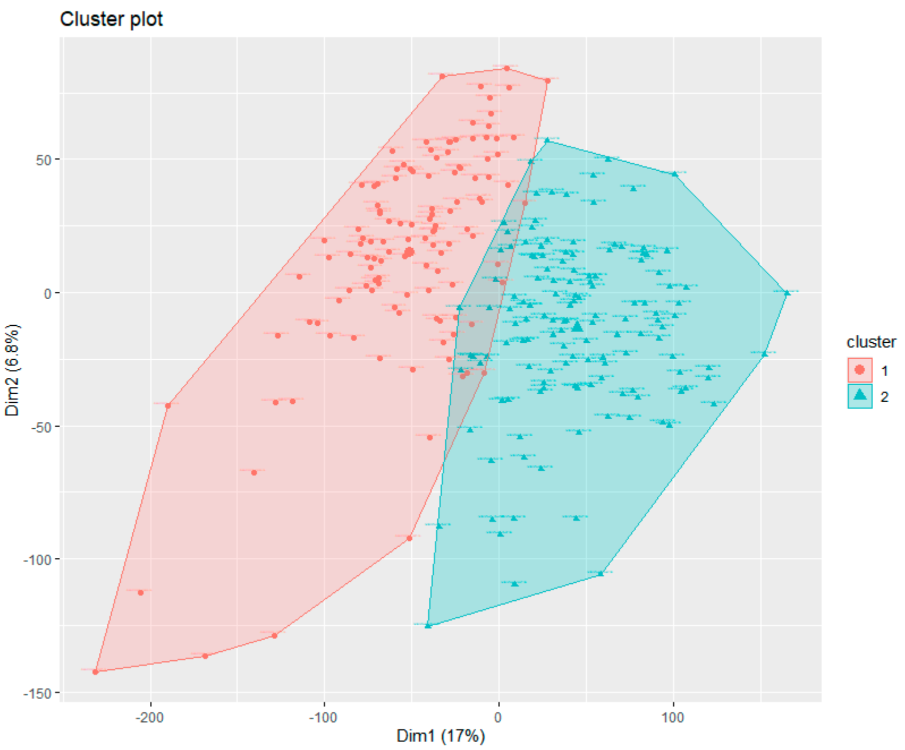

Figure S2: PCA score plot of the first two PCs of the dataset. Clusters plot obtained with the k-means algorithm (k=2) are plotted in different colors inside the first two principal components.

Table S1: Number of samples within each cluster (left). Contingency matrix between the original labels and the k-means cluster labels (right).

Cluster	# Sample per cluster
1	120
2	137

	Cluster 1	Cluster 2
**Normal + Peritumoral**	100	5
**HCC**	20	132

Lists of the twenty communities with the best HCC-control classification performance.

In the following section, we provide a list of the genes in each of the twenty gene communities with the best HCC-Control classification performance, where symbol "///" indicates that there is more than one entry for that specific probe.

Comm_8: NLRP11, RSPO2, FAM13C, TTC30B, LOC100507564, LOC439911, RP11-111J6.2, LOC101929747, LOC646736, DSP, SPARCL1, SLC16A1, AHR, TBC1D4, IFIT5, RAB11FIP2, FABP4, TCEAL1, TK2, FGF2, ICK, CLUAP1, NMT2, CTNNA2, ZNF175, PCP4, DLEU1, DENND4C, FIGF /// PIR-FIGF, PDLIM1, DDX17, FBXL5, AMACR /// C1QTNF3-AMACR, CD36, INSIG2, GNAI1, MINPP1, FTO, HIST1H2BD, NFATC3, NUDT4 /// NUDT4P1, TBC1D9, APBB2, KANK1, NEK3, SNAI2, TMCC1, TTC30A, FAM169A, YPEL1, MYCL, KANSL1L, HIST1H2AC, TOX3, WDR59, CCDC53, RAPGEFL1, MORC4, CAAP1, AEN, PRR5L, THAP10, SUGCT, TBX3, CARF, ERMAP, NUBPL, SLC12A1, CCDC170, PDPK1, FAM117A, MARCH8, SH3BP4, RP11-499E18.1, AXIN2, MED31, BBS2, RNLS, MPLKIP, FAM84A, ANKRD50, CAB39L, TBCK, FAM160B1, ZNF830, ZNRF3, LOC283070, TTC32, PAPD5, RNF217, KIAA1551, ITFG1, OSCP1, ZNF689, STON2, CELF6, LINC00476 /// LOC101930476, SUGT1, TFB1M, DISP1, RBM43, LOC643072, GID4, ZNF319, SCRN2, WLS, PITPNA-AS1, RNASEL, RP1-193H18.2, ACSL6, RAP1GDS1, RP11-355B11.2, GPR155, CTTNBP2, CDADC1, SETDB2, ASB4, RP11-180N14.1, LOC257396, AMACR, TMED6, NPY6R, RP4-813F11.4, KCNU1, CREBRF, PHKB, ATP6V1G1, CPPED1, BRWD1-IT2, DOK7 /// LOC102724043, C21orf37, METTL14, LOC100507557, LOC102724312, TSHZ2;

Comm_12: GIMAP1, CARD16 /// CASP1, CCDC141, FCER1G, ARHGDIB, GABARAP, S100A11, BTG1, ANXA1, RCN1, SON, HLA-DPB1, GPNMB, AKR1B1, SH3BGRL, LAPTM5, MTHFD2, RNASE1, SRGN, PLTP, SEC14L1, CELF2, ARL4C, RGS2, PLSCR1, SLC2A3, LEPROTL1, LYN, ICAM1, WIPF1, CXCL8, CTSS, CD97, RGS1, TGFB1, RASSF2, S100A4, SH2B3, CD53, PLEK, FCGR2A, FCHSD2, EMP3, ADCY7, GCA, VCAM1, CYBB, PRKX /// PRKY, TYROBP, GPR137B, ALOX5AP, TMEM243, GLIPR1, FLI1, PMAIP1, SLC25A24, GALC, ALOX5, PLA2G15, KCNQ1, PDE4D, SLC7A7, ACP5, EVI2A, NAIP, PTGER4, TNFRSF11B, MNDA, CSF2RB, HCAR3, LCP2, GPR183, BCL2A1, ITGAM, JAK2, IL18, GALNS, IGSF6, LY96, KLRC1 /// KLRC2, CD209, IL15RA, MSL3, ENTPD1, NLGN4Y, PF4V1, CXCL3, CPVL, S100A11P1 /// S100A11P1, LCP1, IFI16, PECAM1, KRT8, LMO4, ENPP2, TNFSF12-TNFSF13 /// TNFSF13, ARHGEF6, FAM49A, HLA-DRB4, SLC4A7, C3AR1, SOCS1, PLA2G4A, TLR1, TNFAIP8, MAB21L2, TNFSF13, CD86, PDLIM4, CD44, KCTD12, PTPRC, HLA-DQA1 /// HLA-DQA2 /// LOC100509457, EPB41L3, ANKS1A, DYRK4, DOCK2, ATP10D, CLIC2, ITGA4, TUSC3, IQGAP1, NPHP4, LDHB, RNASE6, ICAM2, FABP3, ARMCX6, MSR1, FAM153A /// FAM153B /// FAM153C /// LOC100507387 /// LOC101928349 /// LOC101930363, BEX4, PTGS1, TMSB4X, CXCR4, MILR1, RAB31, RNASET2, SNN, NAGK, GALNT7, LPAR6, HECA, TMEM51, RPP25, DOCK10, C3orf14, HPSE, C1orf54, MARCH1, MAP7D3, HS3ST2, ZNF586, TM6SF1, CLEC4A, TLR7, SAMSN1, FAR2, NECAP2, LAT2, PTPRE, SLC2A14 /// SLC2A3, AMIGO2, KCNMB4, NABP1, CXCL16, TNFSF13B, RGS18, EMILIN2, CARD6, COTL1, CMTM3, FAR1, PPP1R18, GNG2, LINC01420, DNAJA4, NCOA7, DOCK8, AL832909 /// LINC00984, GLIPR2, GNB4, RCSD1, HENMT1, METRNL, MITF, SYK, SYT13, TTC7B, FMNL2, SUSD1, CHST11, OSMR, ANKRD44, SLFN11, OGFRL1, SPRED1, DOCK11, ZNF518B, NFATC2, FYB, RNF166, LPCAT2, PHACTR2, RILPL2, GIMAP7, LOC101927933 /// LRRC8C, CHIC1, GGTA1P, ODF2L, ZDBF2, SLC6A6, REL, GIMAP6, TLR8, PIP4K2A, TAGAP, ZNF438, LPAR5, RP11-173M1.8, FPR3, ST8SIA4, AL832909 /// LINC00984 /// LINC00984 /// LINC00984, GPRIN3, HAVCR2, RP11-539I5.1, PLEKHA2, TRERF1, NCKAP1L, HLA-DRB1 /// HLA-DRB3 /// HLA-DRB4 /// HLA-DRB5 /// LOC100996809, PIK3CG, KBTBD8, CHSY3, PARP8, ELF4, PPP3CC, MAFF, STK10, TMEM231;

Comm_14: SNX21, COL1A1, HK1, ACTA2, IGFBP7, HTRA1, VIM, COL6A3, APOD, HSPG2, MYOF, EFEMP1, PLAT, MGP, CTSH, COL1A2, CYP1B1, FBN1, CLDN7, STMN2, THBS2, DOCK1, RCAN2, LOXL1, FRZB, HEPH, F2R, SFRP4, TPM2, EDNRA, CCDC85B, LTBP2, CRIP1, LRRC17, ITGBL1, PDE10A, COMP, FGF7, CDH11, ACKR1, PTRF, CRIP2, AQP1, MCAM, TUBA1A, SYT11, MALL, HDGFRP3, CD200, PDLIM3, TGFB1I1, LPHN3, FAP, POSTN, COL3A1, HOPX, IGFBP5, COL4A2, COL5A1, RFTN1, JAM3, CEMIP,

PRKCDBP, HEG1, OLFML2B, CLIC5, TBX2, DKK3, ITGA8, NOV, LOC728392, GREM1, NES, LXN, ARAP3, CACNA2D3, DKK2, PRR16, CCDC102B, PIK3IP1, OGN, SMOC2, UACA, C1QTNF5 /// MFRP, SEMA5B, MGARP, TTYH2, ANTXR1, CYTH3, CCDC80, COL12A1, PARM1, COL8A1, PRSS23, NRARP, PRRX1, GJA5, HEYL, MOB3B, FNDC1, ACVRL1, ADAMTS12, LINC01279, GLT8D2, TMEM119, ZBTB46, SUSD2, LOC102725271 /// NTM, GPX8, MBD5, CDC42EP5, LAYN, PAX8-AS1, RUNX1T1, SHISA3, DCLK1, FMNL3, RP11-134G8.8, GANC, WNT2B, SLFN5, TMEM171, LOC643733, RFTN2, CALHM2;

Comm_15: SH3KBP1, CARD8, PTPRM, RP11-389C8.2, SPARC, GJA1, VWF, LAMA4, SPOCK1, PIK3R3, CD93, CHST15, ITPR1, ITPKB, LMOD1, NID2, GNG11, LMO2, SLCO2A1, TIE1, CDH5, KIT, TRIL, RASGRP3, KL, DISC1 /// TSNAX-DISC1, ARHGAP6, GPR4, CALCRL, KCNJ2, MEF2C, CDC42EP3, PLA2G4C, VEGFC, MDFIC, SPIN2A /// SPIN2B, COL4A1, TCF4, SPRY1, LOC100996668 /// ZEB1, RPS6KA2, GPR116, ZNF423, DOK5, MKL2, LHFP, RGCC, CSGALNACT1, ELTD1, PDGFD, SOX18, PCDH12, APOLD1, MAP3K7CL, COL5A2, ELK3, PHF11, AIF1L, FAM198B, ETS1, CPNE2, ARL8A, ESAM, SASH1, ARHGAP31, PLEKHG1, CLEC14A, SH2D3C, ZNF521, LRCH1, BMP2K, FAM101B, TMTC1, CNOT6L, FAM43A, EPHA4, EMCN, KIAA1211L, BCL6B, ECSCR, CRIM1, CYYR1, PREX2, GUCY1A2, FAM162B, JAM2, PARP11, P2RY8, SHE, TM4SF18, FRMD3, FILIP1, ZNF561 /// ZNF562, RHOJ, CAMK1D, MMRN2, SCN4B, C3orf70, MAML3, GJA4;

Comm_16: GALNT18, KLF7, SEPW1, RP11-309G3.3, RP11-305O6.3, VCL, MMP2, ITPR3, DPYSL3, SCRN1, TNC, GAS7, VASP, PIEZO1, SPINT1, FBLN1, PDLIM7, MFAP2, TPBG, AFAP1, GPRC5B, PROCR, FZD7, SSBP2, SEMA3C, CNN1, SCG2, VDR, MMP7, COL11A1, COL16A1, FZD1, PLLP, PDE4A, MAMLD1, GFPT2, KAL1, MN1, HS3ST1, CHST1, BDKRB2, COL10A1, PTX3, LEFTY1, SI, OXTR, UGT2B17, ANXA13, TUBB6, PLAGL1, LOC100506403 /// LOC101928269 /// RUNX1, MOXD1, CHST3, FUT4, VILL, PMP22, TLR5, FZD2, COL13A1, FCER1A, GPR56, JUP /// KRT17, SULF1, GRAMD1B, AHNAK2, CDR2L, RAP1GAP2, TMEM158, HSPA6, STX2, MUC1, XYLT1, BEAN1, GPR161, CDH6, AES, EXD2, LMCD1, GALNT12, PCYOX1L, HOXC10, ITIH5, OSBPL10, LRRC49, C1orf116, FJX1, ADAMTS5, LINC00312, SFXN3, LBH, VCAN, COL8A2, PMEPA1, FAM129B, LDOC1L, TSHZ3, PAPPA, SPIRE1, KIRREL, RASA3, SEMA6A, RBMS2, MSRB3, ARRDC2, B3GNT9, FIBIN, PLXDC2, LRRN1, UNC5B, SSPN, LOXL4, PLEKHH2, KIAA1211, TSPAN2, C15orf52, IL17RD, ZNF827, ABLIM2, CLDN11, HOXB3, NHS, IL17RA, FOXS1, WISP1, EVC2, SLIT2, ICA1L, PIWIL4, KRT80, VEPH1, RUNX2, HIVEP3, SHC4, PRSS35, RNF145, HOTAIR, FLRT2 /// LOC100506718 /// LOC102724348, RASSF9, SLC26A9, CX3CL1;

Comm_17: KIAA0895L, DPYSL2, YBX3, CTBP2, ANXA4, LAMB1, TP53, ADD3, AFF1, MYCBP2, WWTR1, KIAA0247, CHSY1, NCK2, CAV2, BCL2, ADARB1, VASH1, ENTPD4, LRP3, GRK5, RAPGEF5, KIAA0753, SMAD7, EML1, RECK, TEP1, ZKSCAN7, UBE2I, DYRK1A, CTGF, TNFRSF10B, OSBPL1A, CDR2 /// LOC101060399, CBLB, RIN2, PTCH1, FSCN1, CHMP7, GLYR1 /// SEPT6, ADO, STK38L, WDFY3, AKT3, ARID5B, ABCA2, OBSL1, CAMTA2, FBXL14, IQCK, DDX52, TTBK2, PCGF2, ANKRD36, APP, JAG1, FBXO34, MAGEH1, LTBP3, SPRY4, TNS1, CDK17, PDP1, CCDC82, DNAJC27, ZAK, CHST9, ZNF559, PHF10, NEO1, TMX3, TANC1, IGF1R, TWSG1, TMEM87B, AMOTL1, FRMD6, SH3RF1, ZBTB4, MYADM, RASSF8, PRICKLE2, PRKCE, FAM133B /// FAM133DP, FUT11, HOOK3, RELL1, C20orf194, SLCO3A1, SETBP1, KATNAL1, CEP85L, ACTR3, PCM1, L3MBTL3, GLIS3, TIMP2, RP11-38P22.2, IKZF2, FER, DSEL, TTC17, MAML2, HMCN1, SMARCE1, MGC70870, CYCS;

Comm_23: ATP6V1E2, IQCD, IPP, LINC00847, ATP6V1E2 /// FLJ41757, RP11-533E19.5, IK, TMEM109, COPS6, TTC1, SAR1A, MIR7703 /// PSME2, UBE3C, PRKAB1, PARP4, CYB5R1, SHFM1, RFXANK, ZMPSTE24, SSX2IP, KATNB1, ALG8, ELP4, HSPB11, WWP2, CDK5, ELMO1, TMEM186, POP5, PIGF, SNX15, RPP38, FLAD1, DUS4L, POLR3G, CSRP2, PIR, CUL1, CASK, RNPEP, OSER1, MDH2, CETN2, RNF113A, BBS9, ZNF174, PEX2, TRIM27, MAP2K5, BBS4, COG7, RPP40, APITD1 /// APITD1-CORT, ADIPOR1, YKT6, EMC7, CUEDC2, AMZ2, FN3KRP, TTC4, TMEM14A, CCDC51, ZMAT5, ZSCAN32, BRF2, MRPS17, PLGRKT, TMEM160, ATRAID, PRMT7, NSUN3, PALB2, ALG6, GLRX2, ANKEF1, COA4, IQCG, C7orf25 /// PSMA2, ADCK2, DALRD3, REPIN1, EXOSC1, CCDC113, RPAP2, ZC3HC1, MFSD11, SLC25A33, TBC1D7, PRICKLE4 /// TOMM6, HDAC8, MTPN, DPY30, MRPL45, MON1A, MRPL10, ZMAT2, RPUSD3, METTL23, ISCA2, MANEAL, C12orf45, C17orf58, APTR, COG1, ZNF252P, GPATCH11, LOC102606465, LOC100506100, NCK1-AS1, DUSP28, CHCHD4, C15orf61, KIF9, MIF4GD, STYXL1, PPP4R4, BCDIN3D, WDR92, SCAMP1-AS1, RFESD, LOC100506922, HARBI1, TMEM64, PGAP2, MROH7-TTC4 /// TTC4;

Comm_24: MAB21L3, LOC100507516, OSER1-AS1, BC040901 /// CTC-241N9.1 /// LOC100996419, ZNF501, LOC101927648, SAMM50, SMARCD2, RABEPK, ACTN2, HIRIP3, CDH13, PTPRG, DYNC1I1, PAGE4, BIK, REG3A, MMP3, SLC22A4, FHIT, DKK4, MGAT4C, DUT, PPAP2A, EIF2B4, EPHB2, MID2, VLDLR, NAT6, PRUNE, TTC9, TAF6L, BHLHB9, HIST1H2BJ, SPRYD7, RXRB, ITGA6, GLUL, HAUS4 /// MIR4707, ASPSCR1, NHEJ1 /// SLC23A3, DET1, ODAM, FAM184A, IQCH, BCAS3, CTNNBL1, RPUSD2, ENPP3, HSDL1, STRIP1, ORAI1, MORN2, ZNF777, OSR1, RP5-1136G13.2, TMEM169, LOC643085, FBLN7, C12orf60, FGF14, ULK4, HABP4, ARHGEF28, LINC00348, NECAB1, DTD1, CCDC58, USP46-AS1, ANKRD29, FAM21EP /// FAM21FP, C3orf67, DACH2, LOC101928076, LOC440982 /// ZIC1, LOC100505592, DEFB132;

Comm_27: LINC00152 /// LOC101930489, CCRN4L, SPATA6L, RASGEF1B, BTBD19, KIFC3, AC017002.2, BRE-AS1, MCL1, PHC2, TGM2, STOM, THBS1, BHLHE40, TERF2IP, PRNP, ETS2, JUN, JUNB, ZFP36, PNP, SGK1, CD55, PLK2, LDLR, SDC4, LOC101928916 /// NNMT, CDKN1A, NR4A1, MYC, PFKFB3, SERPINE1, ATF3, S100A8, KIAA0040, IL4R, HES1, EPHA2, NFIL3, PLA2G2A, HMOX1, JUND, HBEGF, IRF8, TSC22D2, TPST1, STAB1, FOSL1, NR4A2, PTGS2, BCL3 /// MIR8085, PLK3, MBNL2, IL6, AREG, ABLIM3, CRP, S100A12, SERPINB8, NRG1, IL18R1, CXCR2, GK, IL1RL1, P4HA1, CREM, TNFRSF1A, TNFSF14, SIK1, IRF7, SAA1 /// SAA2 /// SAA2-SAA4, ID1, PNRC1, FOS, PIM1, GADD45B, DUSP5, CXCL2, AKAP12, CYR61, LBP, C1R, FAM102A, GRAMD4, PXDC1, CEBPD, KDM6B, ID2 /// ID2B, SAT1, SAA1 /// SAA2, CLK1, MIR22 /// MIR22HG, LOC100129518 /// SOD2, LOC100996792 /// MAP2K3, RP11-548H18.2, ARMCX3, PHLDA1, MIR6732 /// ZC3H12A, FOSL2, IPPK, FLRT3, SH3TC1, CHIC2, RAB20, SPSB1, SLCO4A1, ZC2HC1C, SLC38A2, RIPK4, BACH2, KLF4, SLC25A37, SLC35C1, SERTAD1, RASD1, ACTRT3, STK40, PIM3, FAM46A, KDM7A, CSRNP1, ELMSAN1, MAFK, CCDC71L, RFX2, SGMS2, MOB3C, TNFRSF10D, TUSC1, ARGLU1, SOCS3, GAREM, SPIDR, MIR29B2 /// MIR29C, LINC-PINT, USP2, RP6-99M1.2, CA13 /// LOC100507258, NNMT, AK025288, SETX, CREB5, PROK2, SNORD89, MTHFD2L, NAMPT, TRIM15, PPP1R15A;

Comm_28: FAM9B, C14orf37, VNN1, LINC01146, SOD2, NCOR1, RAB14, MLF2, NFKBIA, ID2, EGR1, FURIN, IER2, KLF10, PCOLCE, C3orf62 /// MIR4271 /// USP4, INPP1, SLPI, BAZ2B, DAPK1, SATB1, MAP3K5, SPRY2, GCH1, TLE2, DPYD, INHBB, FES, SDS, SLC3A1, HABP2, C9, CHST7, AKT1, BLNK, GOT1, DUSP6, SYNE1, IGF2 /// INS-IGF2, MGLL, GGTLC1, HPGD, NCALD, ZFP36L1, FBXO21, RND3, NAB2, CCDC69, FRMD4B, TIAM1, DOPEY1, ATG2A, F2RL1, PDLIM5, ADAMTSL3, EPOR /// RGL3, GGT1 /// GGT2 /// GGT3P /// GGTLC1 /// GGTLC2 /// LOC100132705 /// LOC102724197, ALPL, CYFIP2,

SSTR2, HAL, A2M, MPC1, MLPH, C14orf159, NAV2, C8orf4, SLC8B1, INTS6, PLSCR4, POLR1E, PPARGC1A, TSPAN12, TMEM45A, C14orf105, CCDC68, HAMP, VNN3, RAB33B, SLCO4C1, PPP1R3B, FBXW7, SNX9, CMTM6, NFKBIZ, IRF2BPL, CAPS2, TNKS1BP1, ELOF1, SLAIN1, C21orf91, ZSWIM6, MCC, SSU72, FNIP2, TDRP, FAM110C, WDR72, SOX6, USP9Y, ANTXR2, SLC45A3, SORCS2, SRSF8, KIF12, BC022047, CLRN3, HSPA5, SERPINA1, FAAH2, IP6K3, LOC100505985, GPR180, TTC39B, MBNL2 /// MBNL2, MASP1, RP11-469M7.1, EBF4, RAB27A, SMIM6, SAMD12, EIF5 /// SNORA28, RASSF8-AS1, SAMD5, NPW, MTCP1, AGPAT2, MYO7A, TJP3, AQP3, C11orf24;

Comm_29: C1orf210, ADAMTS9-AS2, LOC100996760, FLJ35700, CSRP1, ENC1, QSOX1, KRT19, EPCAM, MYO10, LTF, SFRP1, TACSTD2, PRSS8, FMOD, LTBP1, GPC1, HEXIM1, AMOTL2, PTPRN2, ANXA8 /// ANXA8L1, PDGFRA, GALNT3, DDB2, RBP1, SCNN1A, FGFR2, WFDC2, SH3YL1, SLC34A2, PTPN13, PROM1, RGS4, F3, EPHB6, GPRASP1, MTMR9, TCF21, SRPX, TGFA, EFNB3, CFTR, GABRP, RARB, AMPH, LIF, CLDN10, BMP5, FXYD2, CX3CR1, KCNS3, SELP, LHX2, NME5, CILP, CXCL6, ANK3, SLC5A1, ADAMTSL2, SMAD6, FXYD2 /// FXYD6-FXYD2, SFRP5, PTGIS, WNT4, CCND1, KRT7, ANXA3, PNMA2, LRRN3, CCL19, KCNJ15, SCTR, DEFB1, SPINT2, NFATC1, TNFRSF10C, FGFR1, CEACAM6, OVOL2, PBX1, ENDOD1, MFAP4, FAM171A1, DPT, TSPYL5, SEMA5A, PDE8B, CDKN1C, MFSD7, OLFML3, RAB25, TMEM242, PLEK2, SLC22A17, LEPREL1, TPPP3, PELI2, MICALL2, ELMO3, EVC, KCNJ16, SMPD3, VTCN1, HS3ST3A1, CHST4, VPS37B, EDN1, THSD4, INMT, SULF2, SCD5, APCDD1, SPNS2, TMEM125, PRICKLE1, ITGB8, AQP4, TMC4, PCDH19, NCAM1, SMAD9, ST6GALNAC1, CLIC6, LGR6, VSIG2, EPB41L4A, SCN7A, NALCN, BHLHE22, CYS1, EFCAB4B, ST6GAL2, SLC14A1, MUM1L1, SPATA18, OVOL1, FAM19A5, TMEM200C, WNK2, CCBE1, GABRB3, RSPH3, PLCXD3, ZNF471, PCDP1, ANGPTL1, NKD2, FAM83B, LRRC4C, SLC28A3, FKBP1A-SDCBP2 /// SDCBP2, TMEM92, ZFP3, PLA2R1, SCARA5, FAT3, PPAPDC1A, GGT6, FAM150B, LOC441528, LOC102724387, PKHD1, ZNF667-AS1, TBXA2R, ARHGAP8 /// PRR5-ARHGAP8;

Comm_30: FILIP1L, GLIDR /// LOC728903, GSN, EMP1, TIMP1, LUM, AEBP1, ENG, GAS6, TRIM2, AKAP2 /// PALM2-AKAP2, SYNPO, GYPC, FHL2, C7, FBLN5, ALDH1A3, DGKA, FSTL3, GUCY1B3, RGS3, LRRC32, THBD, KDR, NRGN, PDE2A, EMILIN1, PRELP, EDNRB, FLRT2 /// LOC100506718, GAS1, GEM, CLDN5, CCL21, RCBTB2, LEPREL2, AOC3, CLEC3B /// EXOSC7, RAMP3, HOXB2, TAGLN, MMRN1, CPA3, WNT2, RAMP2, OMD, FOXF1, EPHA3, EGR3, LDB2, HTR2B, TEK, ITGA10, CH25H, ISLR, ID3, PDE1A, TSC22D3, FSTL1, FAM107A, COL6A2, ID4, RGS16, EFEMP2, CHI3L1, SPON1, NR2F1, MXRA5, TMEM47, GATA2, TGFB3, GATA6, CPZ /// GPR78, CLEC11A, FMO2, DCN, PXDN, CLIP3, ELN, COL14A1, SVEP1, TCF25, COL6A1, NFASC, LAMA2, ERG, APLNR, KANK3, IRAK3, BGN, PLA2G5, TNXA /// TNXB, CCL2, COX16 /// SYNJ2BP-COX16, FXYD6, NPDC1, ASPN, DACT1, TMEM204, KLF2, FAT4, LRRK1, WFDC1, ZFPM2, GAL3ST4, LHX6, SOX17, RASIP1, CRISPLD2, GPR124, AKAP13, DCHS1, ADAMTS1, CYBRD1, PARVA, SDPR, C1orf21, CCDC3, LATS2, ABI3BP, RNF135, DLL1, MIR100HG, ITPRIP, PCDH18, STEAP4, ROBO4, PIGR, PGM5, ADAMTS2, PODN, ARHGAP23, CNRIP1, CPXM2, FGD5, NOSTRIN, PPP1R14A, CCDC146, C11orf96, INHBA, EVA1C, GUCY1A3, CDYL2, ITGA9, PLAC9, CACNA2D1, SYNPO2, TCEAL7, MRGPRF, CPXM1, S1PR3, PRKG1, PEAR1, PCDH7, NRP2, TMEM178A, COL4A4, FLT4, GJD3, USP53, F2RL2, HIC1, PTPRB, HAS2, GALNT16, MYCT1, CCM2L, HECW2, LOC101928612, KIF26A, ST6GALNAC3, RBMS3, IPO11 /// LRRC70, NPR1, MRC2, RCN3;

Comm_31: AGAP2-AS1, RP3-525N10.2, LOC102723918, POLR2A, PRKACA, ADIRF, IGFBP6, LTBP4, LDOC1, COX7A1, MAPK10, TLE4, AHDC1, CKMT2, HOXB6, SCN1B, GGT5, BAI3, TPSAB1, WISP2, HSPB2, NGFR, PTGIR, MLANA, LTC4S, GPR143, ALDH1A2 /// LOC101928635, TPSB2, ADIPOQ, GPM6B, SGCA, PTGDS, TENC1,

PDZRN3, OLFM1, TMEM159, MXRA8, KAZN, SGCD, PINLYP, TPSAB1 /// TPSB2, ZSCAN18, PHLDA3, TINAGL1, SLC24A3, RASL11B, MAP3K6, LAMC3, RAI2, NXN, PCDH9, NPR3, HAND2-AS1, EPB41L4A-AS2, PDZRN4, CRTAC1, NTRK2, DENND2A, C2orf40, C1QTNF7, SEMA6D, PTPRS, ZNF462, CYGB, C8orf58, MIR424 /// MIR503 /// MIR503HG, DCLK2, UNC5C, PHACTR3, DACT3, MIR424 /// MIR503HG, MYZAP, RP11-999E24.3, MAMDC2, CNTN4, CLSTN2, S1PR5, DNM3OS, LINC00472, RNF150, LOC101927263, SMCO3, WDFY3-AS2, LOC100506388, DGKE, RP11-710C12.1, RP11-642D21.1, AP001347.6 /// BC024173 /// BC048201, WDR86, CASKIN2;

Comm_32: RTP3, C9orf43, SERPINC1, RAB11B-AS1, MROH2A, RP11-6F2.5, EP300-AS1, HNF4A-AS1, ANXA6, GRHPR, HSD17B4, ALDH2, SORD, EPHX1, SERPINF1, GCLC, ETFB, CRYZ, IFIT1, CYP27A1, FAM47E /// FAM47E-STBD1 /// STBD1, MAOB, SUOX, MGST2, AMT, CYP11A1, MAOA, GSTM2, PC, UGT1A1 /// UGT1A10 /// UGT1A4 /// UGT1A6 /// UGT1A8 /// UGT1A9, GSTM1, ACOT13, GJB1, HAGH, MPDZ, GCAT, MAP3K14, ALDH1L1, EHHADH, DEPDC5, ACADSB, ACOX2, HSD11B1, OCEL1, AQP9, ALAS1, C4BPA, MTTP, BPHL, JAKMIP2, ARSE, AADAC, HPD, ABCC2, FMO4, SULT2A1, FMO3, LIPC, CPB2, APOC2 /// APOC4 /// APOC4-APOC2, HDAC6, SALL1, BAAT, CCDC106, NR1I3, NCRNA00185 /// TTTY14, SLC17A2, CCL16, CYP7A1, LECT2, CA5A, FDXR, PKLR, C4BPB, HPR, POR, RARRES2, BAIAP2, TST, CES1 /// LOC100653057, CES2, NTHL1, ABCG2, SLC9A3R2, DHTKD1, LPA /// PLG, ABCB1 /// ABCB4, RORA, HYAL1, KCNB1, ABCB11, BDH1, PEBP1, PHF8, ABTB2, HSD17B8, ATP6V0E2, SELENBP1, HLX, CDK18, APOM, NECAB2, RUNDC3B, ABCA6, CYP2C9, DCXR, ECI2, TSKU, SLC25A10, LHPP, DYSF, PCTP, SYBU, ACSF2, CERS4, TMEM140, THNSL2, MACROD1, ETNK2, SLC47A1, LIME1, PLEKHF1, DECR2, SLC27A5, ANGPTL3, CDHR5, HAO1, CRYL1, ALDH6A1, TMEM143, C16orf70, ADH4, APOA5, HINT2, ABHD14B, GPT2, KLC4, AR, TCEA3, DEPDC7, ATF7IP2, HFE2, DQ570096, NAGS, LINC00324, SEC14L2, LOC101928230, TMEM52, HNF4A, CTD-2325A15.5, GLYAT, DMGDH, STARD10, CYP8B1, C7orf13 /// LINC01006, ATP1A1-AS1, LOC149703, LOC102723493, HGD, RP11-21L23.2, LAMTOR5-AS1, RP11-736K20.5, C5orf27, LOC101927609, RP11-740C1.2, RP11-384L8.1, ACSM2A /// ACSM2B, ACOT6, LOC101929880 /// QPRT, LAMA5-AS1, PRAP1, LOC101929036 /// PAH, ACSM5;

Comm_33: KLHDC1, C12orf66, MGC32805, ZNF385B, BC038731 /// CTD-2284J15.1, FTCDNL1, CHADL, LOC284014, ALDH1L1-AS2, RIPPLY1, LOC100506368, C7orf49 /// LOC653739, RBM26-AS1, AADACP1, BLCAP, MTHFD1, ITPR2, C16orf62, THOP1, PPL, FAM8A1, MME, KLF9, GSTT1, KAT2B, DMD, S1PR1, IFT88, HLF, AOX1, UST, MYL5, SLC16A4, PER2, KCNJ8, SLC22A3, CYP1A1, PLIN1, MYH3, CYP3A4, SLC6A12, ECM2, ADRB2, HERC3 /// LOC101929134, DSG1, SULT1B1, LRP2BP, EFHD1, XPC, CYP2E1, GPER1, SEPT4, CLDN14, DAAM2, STARD13, SS18L1, EXPH5, MYRIP, PACRG, AASS, TSC22D1, LINC01314, MICU1, BACE1, ACSL5, RWDD2B, LZTFL1, BBS1, TCTN1, PACSIN3, C11orf71, RNMTL1, SIRT5, SCUBE2, TMEM100, FLVCR2, CA14, SH2D4A, C16orf95, LGSN, ELF5, ACE2, THNSL1, NEU4, EBPL, BOK, CLDN2, ZMYND12, FBXO31, FAM210B, SH3D19, SH3BGRL2, SLC1A2, CYB5D2, UBE2QL1, MIB2, PANK1, C7orf55, ZNF18, KLF12, TRAM2-AS1, GFRA1, SFXN2, KLHDC9, IDNK, RAB37, SENP8, GALNT15, LOC102723864, GPR146, HNMT, GNG7, SHF, NADK2, LINC00959, FAM120C, TMEM150C, TPRG1, WDR78, KANSL1-AS1, LINC00844, RP11-320N7.2, TENM2, ARSD, FLJ22763, C5orf55, VSTM4, RASL10B, SSTR1, FAM162A, RP11-635N19.1, SLC16A10, SHH, RP11-295M18.6, LINC00238, PDE11A, RP11-477N3.1, HEPACAM /// HEPN1, MAN2C1, SMIM2-AS1, UBXN10, HOGA1, FLJ37035, TRPM7, C15orf43, CRLS1, POLN, VWCE, LOC100130476, SLC2A12, LOC101928370;

Comm_35: TIMD4, CLEC12A, NLRC4, SIGLEC11, SH2D1B, SPIC, GLIDR, B3GNT7, WDR17, SIGLEC16, SDC3, FBN2, CD68 /// LOC101928634 /// SNORA67, CD4, PRKAR2B,

SNCA, VSIG4, FOLR2, SPI1, CFD, BST1, RELN, HK3, CD33, BMP6, CD180, HPGDS, LILRB5, EMR1, SIGLEC7, CD226, NPY5R, IL10, CD160, KLRG1, LILRA1, LILRB3, SH3BP2, DENND3, DOK2, RASGRP2, SLC7A8, TRDV3, DOK1, ZNF816 /// ZNF816-ZNF321P, BEX1, DPEP2, TOR4A, SIGLEC5, P2RY13, CD244, ACKR4, ADAP2, TNFAIP8L2, ABI3, FAM167B, ADORA3, HVCN1, SLC9A9, NAPSB, C2orf88, KCNJ10, CMKLR1, MIR6125 /// USP15, NRROS, P2RY12, NFAM1, CR1, GPR82;

Comm_40: TMEM53, LINC01128, LOC101928303, LOC339874, RP3-400B16.4, SOD1, LRP1, ECH1, SDHA, ECHS1, SCP2, ARAF, BCKDK, UQCR11, PEPD, BLVRB, HSD17B10, BCKDHA, PDK2, SDHB, ABHD14A-ACY1 /// ACY1, HMGCL, DDT /// DDTL, FPGS, APOE, SLCO2B1, PEX14, CCS, PCBD1, ALDH5A1, CTSF, SLC25A20, TPMT, ALDH4A1, GADD45A, ETHE1, NDUFAF1, GMPR, SURF1, NAGLU, ENDOG, GCHFR, ICAM3, SH3BGR, ZBTB48, CYP2J2, PAFAH2, GSTT2, CRYM, OASL, ATRIP /// TREX1, ACADL, CYP4F11, ADH1C, GPT, ACYP2 /// LOC101927144, TAT, OTC, SLC17A3, INHBC, HLCS, TFR2, UGT2A1 /// UGT2A2, DGCR6 /// DGCR6L, GCDH, ABCC6 /// LOC101930322, UGT1A1 /// UGT1A10 /// UGT1A3 /// UGT1A4 /// UGT1A5 /// UGT1A6 /// UGT1A7 /// UGT1A8 /// UGT1A9, COMT, ALDH7A1, QDPR, GLRX, EPHX2, CRAT, GSTZ1, ECI1, CRADD, MIR6778 /// SHMT1, SARDH, AKR1C4, CDK10, C21orf33, SRD5A1, KHK, CYP4A11, HADH, NDUFS7, PMPCA, GRPEL1, CEBPB, PCCB, ATP5D, ZNF688, STARD5, CYB5A, SMPD1, AKR7A3, DCAF11, EI24, AZGP1 /// AZGP1P1, PRAMEF10, SLC37A4, GSTK1, VKORC1, MSRB1, DHRS4 /// DHRS4L2, RMDN3, ROGDI, FAHD2A, PNPO, NIT2, DAK, COQ6, FCGRT, ASB13, METRN, PXMP2, RBKS, MRPL46, ECHDC3, DHX58, C1orf50, COPZ2, WDR25, SLC2A9, LONRF3, RIC3, HHLA3, ROPN1B, TMEM176B, GFOD2, PECR, UGT1A1 /// UGT1A3 /// UGT1A5 /// UGT1A8 /// UGT1A9, SIRT3, SPSB3, IL17RC, VRK3, SLC25A15, C11orf1, AGMAT, PDXP /// SH3BP1, ALKBH7, TXNDC11, RBP5, ACTR3C, AKR1C6P, TPRG1L, TMEM205, ZNF511, OCIAD2, SMIM12, DHRSX, MRPL54, MMAB, MMP24-AS1, ABTB1, TANGO6, TSTD1, LRTOMT, CISD3, ABHD15, N6AMT2, SUSD3, OXNAD1, CCDC57, UQCR10, ZNF584, USP30-AS1, NUDT7, PRKAG2-AS1, LDHD, CTD-2256P15.2, ACOT4, LINC00526, SETD3, FUOM, ACY3, GPRC5C, ANKRD35, ABCG8, GGACT, CDH23 /// LOC100653137, SELO, XXbac-B476C20.9, LOC101929857 /// LOC101929864 /// SLX1A /// SLX1A-SULT1A3 /// SLX1B /// SLX1B-SULT1A4 /// SULT1A3 /// SULT1A4, MPND, ESPN, TM6SF2, TMPRSS6, C14orf182, HORMAD2, ENPP7, ANKS4B, DPH6-AS1, RP11-295P9.12, LOC101927287, PEX11G, ASMTL, SIGIRR;

Comm_41: GPR182, C9orf66, GPR128, TCTEX1D1, WDR66, LOC389906, CTB-167B5.2, LOC389906 /// LOC441528, PRR26, CLEC4G, LOC101927668 /// MACC1, LINC01093, SAMD11, ZBED3-AS1, PP12708, SPAG7, CD81, ACADVL, SARAF, OAT, SH3BP5, LY6E, TSPAN7, FOSB, IL1R1, SOCS2, FEZ1, WASF3, PLCB2, MT1X, MRC1, TGFBR3, MT1G, VIPR1, DEFA1 /// DEFA1B /// DEFA3, EGR2, CCL14 /// CCL15-CCL14, NPY1R, GSPT2, DNASE1L3, AKAP7, CD1D, SKAP1, MARCO, MYOM2, SLC18A2, FCN3, PDE4DIP, PTH1R, CRHBP, ASPA, IL13RA2, CETP, SELE, CFP, CYP26A1, DBH, MT1H, LRRC6, SLC16A5, CD5L, TXK, CDH19, SCARF1, COLEC10, FCN2, LILRA2, CITED2, ESRRG, CLEC4M, CBFA2T3, NDEL1, RCAN1, HBB, PLIN2, MYD88, PEG3, TFPI2, ECM1, GPM6A, APOL1, PLA2G16, CXCL12, ZNF330, IL33, NR4A3, HGF, KCNAB1, FHL1, V9415, CCL23, TNFSF11, FCGR2B, MT1HL1, HBA1 /// HBA2, IGFBP3, MT2A, FYN, SPG20, AIM1, TRANK1, CTSB, HBG1 /// HBG2, CAND2, MT1F, PAMR1, CDC42EP2, PTPRD, MEIS3P1, PPBP, MTUS2, DIRAS3, MT1E, MT1M, DHX32, AVPI1, MAN1C1, EHD3, PLAC8, LYVE1, TRPV4, COLEC11, GNA14, NXF3, ANKRD55, STAB2, CDHR2, CYB5R2, LOC101927965 /// LRAT, NDST3, CLEC1B, KLRF1, C1QTNF1, KCNMA1, DPH1 /// OVCA2, CXCL14, ARRB1, NTN4, ITLN1, TMEM27, ADAMTS13, HOTS, H19 /// MIR675, TMEM261, MRPS6, MFSD2A, LIFR, UBE2E2, RNA45S5, FAM229B, LONRF1, JDP2, DNALI1, LOC100506870 /// LOC283140, NAAA, SLC30A4, MAGI2-AS3, FAM65C, RGAG4, RARA-AS1, NAP1L5, RSPO3, ARHGAP20, PNPLA7, YTHDC1, CPED1, CD82,

DACH1, CAMK4, KANK4, ADRB1, ZNF502, LRRK2, INTS10, GRAP, CNTN3, BMPR1B, PITPNM3, PDE7B, PCAT19, HHIP, EPHB1, OIT3, COL6A6, FREM2, DDX19B, SLC6A19, MRO, RXFP1, SMIM24, BCO2, SLITRK6, PARP6, PPM1K, BDH2, FAM83F, VMO1, ZNF597, LOC100506990, SLC19A3, TLR3, DCUN1D3, NCKAP5, FOXO6, RWDD1, PRAM1, BMPER, SYTL5, RNF180;

Comm_42: ERICH5, HDX, NUB1, MSN, ANXA5, PALLD, HNRNPAB, SOX4, ARPC1B, STXBP1, ITGAV, TES, GBP2, GPX2, PMM2, F13A1, RTN1, FIG4, BLVRA, TSPAN8, NFKBIE, FZD6, KDELR3, LGALS4, KIAA0513, ADA, ANKRD6, FGF13, RELB, BTN2A2, CBR3, SPA17, CCL20, PLAU, PCSK5, ANGPT1, GABBR1 /// UBD, MICB, HCG4, TNIP1, CD24, CAPN2, ANXA2P2, ATP1B3, LGALS3, SWAP70, RIPK2, SPP1, NCF2, IFT20, ANXA2, BIRC3, ANXA2P3, CD58, RHOQ, GALNT10, LCN2, RRAS, SACS, PHLPP2, CEP112, RAP2B, CRYBG3, SP140L, GOLM1, BAG3, SCPEP1, SNX24, CADPS2, PDZK1IP1, GTDC1, UTS2, EOGT, IL27RA, WDR13, ACER3, STARD3NL, EPDR1, MESDC1, TRIM6, CNTNAP3 /// CNTNAP3B /// CNTNAP3P2, ASAP1, C12orf75, EDIL3, SLC39A10, MFSD6, HMGB3, PAG1, CTHRC1, LINC00152 /// LOC101930489 /// MIR4435-1HG, FBXO32, NCEH1, TMOD2, CLIP4, PDK1, BCAT1, FAM171B, MBOAT1, HKDC1, MYO5A, GPR176, ADPRH, SLC44A3, PCDH17, ADTRP, EHD4, TMEM45B, SGK223, PLCXD2, CDKN2B, CCL28, TNFRSF11A, C2CD4A, LOC100507507;

Comm_43: CAPNS1, RPS11 /// SNORD35B, PFN1, CD63, NPC2, LITAF, ODC1, SYNGR2, LGALS1, PLP2, ILK, PLEKHB2, GYG1, IFNGR2, EPB41L2, DYNLT1, PLIN3, NUP93, CD2BP2, FABP5, GNPDA1, PTPN1, SLC1A3, DDIT4, HDDC2, STX4, DNASE1L1, RASSF1, SLC2A5, PPP1R3D, EXOSC9, BTG3, SMPD2, CLTB, GLB1L, HYAL2, SERPINH1, VOPP1, CLIC1, ATP6V1E1, ARPC2, TRIP6, RAD51B, RGS13, PFKM, GRN, MCAM /// MIR6756, SERPINB6, SLC25A6, TBC1D1, CHN1, STX7, RNF4, CAMSAP1, JMJD6, MFSD5, GAPDH, CAP1, P2RX5 /// P2RX5-TAX1BP3 /// TAX1BP3, BTN2A1, TBCB, YWHAB, VPS4A, MAP7D1, ARHGAP17, DBNDD2 /// SYS1 /// SYS1-DBNDD2, C17orf62, ATP13A2, DRAM1, TFPT, RNF34, FKBP11, TMEM185B, VANGL1, DNASE2B, TMEM223, ATP1A1, CSRNP2, ELMO2, ACOT9, R3HDM4, FLII, OR2A20P /// OR2A9P, DBNL /// MIR6837, CHMP3 /// RNF103 /// RNF103-CHMP3, TMEM206, TIMM22, ADPRHL2, BTBD10, NT5C3A, VPS18, C17orf49 /// RNASEK-C17orf49, MRAS, ODF2, CTSC, RRP36, C19orf25, KCTD10, TMEM165, MYEOV2, AFAP1L1, STAMBPL1, MVB12A, TMEM185A, AP1S2, PRMT2, SLC7A6OS, ETV6, LOC374443, TRAFD1, RASSF4, MED27, EHBP1L1.

Table S2. Gene Set Enrichment Analysis of genes in Comm_41

Gene Set Name	p-value	FDR q-value
GOBP_TUBE_DEVELOPMENT	4.02E-13	5.49E-09
GOCC_EXTERNAL_ENCAPSULATING_STRUCTURE	1.07E-10	4.89E-07
NABA_MATRISOME	2.75E-10	9.41E-07
GOBP_TISSUE_DEVELOPMENT	1.34E-09	3.27E-06
GOBP_TUBE_MORPHOGENESIS	1.44E-09	3.27E-06
GOCC_CELL_SURFACE	1.67E-09	3.27E-06
GOBP_REGULATION_OF_CELL_POPULATION_PROLIFERATION	2.49E-09	4.25E-06
GOBP_DIGESTIVE_TRACT_MORPHOGENESIS	4.45E-09	6.75E-06
GOBP_EPITHELIUM_DEVELOPMENT	2.28E-08	2.95E-05

GOBP_RESPONSE_TO_GROWTH_FACTOR	2.40E-08	2.95E-05
NABA_MATRISOME_ASSOCIATED	2.59E-08	2.95E-05
GOBP_KIDNEY_EPITHELIUM_DEVELOPMENT	2.90E-08	2.99E-05
GOBP_ANATOMICAL_STRUCTURE_FORMATION_INVOLVED_IN_MORPHOGENESIS	3.06E-08	2.99E-05
GOCC_COLLAGEN_CONTAINING_EXTRACELLULAR_MATRIX	6.30E-08	5.74E-05
GOBP_RESPONSE_TO_ENDOGENOUS_STIMULUS	7.49E-08	6.40E-05
GOBP_EXTERNAL_ENCAPSULATING_STRUCTURE_ORGANIZATION	1.55E-07	1.24E-04
GOBP_MESONEPHROS_DEVELOPMENT	1.85E-07	1.39E-04
GOBP_EPITHELIAL_TUBE_MORPHOGENESIS	1.94E-07	1.39E-04
GOBP_CELL_ADHESION	3.09E-07	1.88E-04
GOCC_PLASMA_MEMBRANE_REGION	3.16E-07	1.88E-04
GOBP_ENZYME_LINKED_RECEPTOR_PROTEIN_SIGNALING_PATHWAY	4.27E-07	2.43E-04
GOBP_ANIMAL_ORGAN_MORPHOGENESIS	6.12E-07	3.35E-04
GOBP_DIGESTIVE_SYSTEM_DEVELOPMENT	9.92E-07	5.21E-04
GOBP_REGULATION_OF_LOCOMOTION	1.13E-06	5.70E-04
GOBP_EMBRYONIC_DIGESTIVE_TRACT_MORPHOGENESIS	1.22E-06	5.94E-04
GOBP_LOCOMOTION	1.31E-06	6.17E-04
GOBP_NEPHRON_DEVELOPMENT	1.38E-06	6.30E-04
GOBP_REGULATION_OF_PHOSPHOLIPASE_ACTIVITY	1.53E-06	6.39E-04
GOBP_CELL_MOTILITY	1.53E-06	6.39E-04
GOCC_INTRINSIC_COMPONENT_OF_PLASMA_MEMBRANE	1.54E-06	6.39E-04
GOBP_GLOMERULUS_DEVELOPMENT	1.83E-06	7.35E-04
GOBP_REPRODUCTION	1.88E-06	7.35E-04
GOBP_POSITIVE_REGULATION_OF_CELL_POPULATION_PROLIFERATION	3.18E-06	1.21E-03
GOBP_MORPHOGENESIS_OF_AN_EPITHELIUM	3.80E-06	1.40E-03
GOCC_CELL_PROJECTION_MEMBRANE	4.44E-06	1.60E-03
GOBP_WATER_TRANSPORT	4.62E-06	1.62E-03
GOBP_REGULATION_OF_LIPASE_ACTIVITY	5.23E-06	1.79E-03
GOBP_MORPHOGENESIS_OF_A_BRANCHING_STRUCTURE	5.52E-06	1.84E-03
GOBP_NEGATIVE_REGULATION_OF_CELL_POPULATION_PROLIFERATION	5.66E-06	1.84E-03
GOCC_APICAL_PLASMA_MEMBRANE	6.62E-06	2.10E-03
GOBP_POSITIVE_REGULATION_OF_DEVELOPMENTAL_PROCESS	6.88E-06	2.14E-03
GOBP_CELLULAR_RESPONSE_TO_ENDOGENOUS_STIMULUS	7.15E-06	2.17E-03
GOBP_POSITIVE_REGULATION_OF_SIGNALING	8.50E-06	2.53E-03
GOBP_EMBRYONIC_DIGESTIVE_TRACT_DEVELOPMENT	9.63E-06	2.80E-03
GOBP_REGULATION_OF_MULTICELLULAR_ORGANISMAL_DEVELOPMENT	1.14E-05	3.21E-03
GOBP_FLUID_TRANSPORT	1.15E-05	3.21E-03
GOBP_TISSUE_MORPHOGENESIS	1.35E-05	3.69E-03

Table S3. Gene Set Enrichment Analysis of genes in Comm_41

Gene Set Name	p-value	FDR q-value
NABA_MATRISOME	2.86E-19	3.91E-15
NABA_MATRISOME_ASSOCIATED	1.32E-14	7.16E-11
GOBP_DEFENSE_RESPONSE	1.57E-14	7.16E-11
GOCC_CELL_SURFACE	7.49E-12	1.71E-08
GOBP_BIOLOGICAL_PROCESS_INVOLVED_IN_INTERSPECIES_ INTERACTION_BETWEEN_ORGANISMS	1.38E-11	2.54E-08
GOBP_OPSONIZATION	1.49E-11	2.54E-08
GOBP_CELL_SURFACE_PATTERN_RECOGNITION_RECEPTOR_SIGNALING_PATHWA	7.40E-11	1.12E-07
Y	1.48E-10	2.02E-07
REACTOME_LECTIN_PATHWAY_OF_COMPLEMENT_ACTIVATION	2.18E-10	2.71E-07
GOCC_EXTERNAL_SIDE_OF_PLASMA_MEMBRANE	4.74E-10	5.39E-07

GOBP_POSITIVE_REGULATION_OF_PHAGOCYTOSIS	6.08E-10	6.39E-07
GOCC_SIDE_OF_MEMBRANE	6.95E-10	6.78E-07
GOCC_SERINE_TYPE_ENDOPEPTIDASE_COMPLEX	1.04E-09	9.49E-07
GOBP_COMPLEMENT_ACTIVATION_LECTIN_PATHWAY	1.47E-09	1.21E-06
GOBP_DEFENSE_RESPONSE_TO_OTHER_ORGANISM	1.50E-09	1.21E-06
GOCC_SERINE_TYPE_PEPTIDASE_COMPLEX	1.84E-09	1.40E-06
NABA_ECM_AFFILIATED	3.77E-09	2.71E-06
GOBP_REGULATION_OF_PHAGOCYTOSIS	7.99E-09	5.46E-06
GOBP_IMMUNE_RESPONSE	1.53E-08	9.92E-06
GOMF_MANNOSE_BINDING	2.32E-08	1.44E-05
GOBP_REGULATION_OF_IMMUNE_EFFECTOR_PROCESS	2.53E-08	1.50E-05
GOCC_INTRINSIC_COMPONENT_OF_PLASMA_MEMBRANE	3.63E-08	2.07E-05
GOMF_SIGNALING_RECEPTOR_BINDING	5.08E-08	2.78E-05
GOMF_CARBOHYDRATE_BINDING	5.45E-08	2.86E-05
REACTOME_INITIAL_TRIGGERING_OF_COMPLEMENT	7.43E-08	3.76E-05
GOBP_INNATE_IMMUNE_RESPONSE_ACTIVATING_SIGNAL_TRANSDUCTION	7.84E-08	3.83E-05
GOCC_COLLAGEN_TRIMER	1.04E-07	4.91E-05
GOBP_ENDOCYTOSIS	1.10E-07	5.00E-05
GOBP_PHAGOCYTOSIS	1.28E-07	5.63E-05
GOBP_HUMORAL_IMMUNE_RESPONSE	1.36E-07	5.82E-05
GOBP_PHAGOCYTOSIS_RECOGNITION	1.72E-07	7.14E-05
GOBP_INNATE_IMMUNE_RESPONSE	2.24E-07	9.00E-05
WP_COMPLEMENT_SYSTEM_IN_NEURONAL_DEVELOPMENT_AND_PLASTICITY	2.97E-07	1.13E-04
REACTOME_METALLOTHIONEINS_BIND_METALS	2.97E-07	1.13E-04
GOBP_DIVALENT_INORGANIC_CATION_HOMEOSTASIS	3.36E-07	1.24E-04
REACTOME_COMPLEMENT_CASCADE	3.64E-07	1.31E-04
GOBP_CELL_RECOGNITION	4.43E-07	1.55E-04
GOBP_RESPONSE_TO_INORGANIC_SUBSTANCE	5.30E-07	1.81E-04
GOBP_COMPLEMENT_ACTIVATION	6.53E-07	2.18E-04
REACTOME_RESPONSE_TO_METAL_IONS	6.75E-07	2.20E-04
GOBP_NEGATIVE_REGULATION_OF_GROWTH	8.15E-07	2.59E-04
GOBP_DETOXIFICATION_OF_COPPER_ION	8.96E-07	2.78E-04
GOBP_CELLULAR_METAL_ION_HOMEOSTASIS	1.02E-06	3.09E-04
GOBP_REGULATION_OF_GROWTH	1.27E-06	3.79E-04
GOBP_ACTIVATION_OF_INNATE_IMMUNE_RESPONSE	1.31E-06	3.81E-04
GOBP_LOCOMOTION	1.41E-06	4.00E-04
GOBP_REGULATION_OF_CELL_POPULATION_PROLIFERATION	1.57E-06	4.38E-04
GOMF_G_PROTEIN_COUPLED_RECEPTOR_BINDING	1.73E-06	4.73E-04
GOBP_STRESS_RESPONSE_TO_METAL_ION		
